# Development of a novel oil-in-water emulsion and evaluation of its potential adjuvant function in a swine influenza vaccine in mice

**DOI:** 10.1186/s12917-018-1719-2

**Published:** 2018-12-22

**Authors:** Jinqiu Zhang, Jinfeng Miao, Xiangan Han, Yu Lu, Bihua Deng, Fang Lv, Yanhong Zhao, Chan Ding, Jibo Hou

**Affiliations:** 1 National Research Center for Veterinary Vaccine Engineering and Technology of China, Jiangsu Academy of Agricultural Sciences, Nanjing, 210014 China; 2 College of Veterinary Medicine, Nanjing Agricultural University, Nanjing, 210095 China; 3 Shanghai Veterinary Research Institute, Chinese Academy of Agricultural Sciences, Shanghai, 200241 China

**Keywords:** Swine influenza virus, Oil-in-water emulsion, H3N2 vaccine, Adjuvant

## Abstract

**Background:**

Vaccination is the principal strategy for prevention and control of diseases, and adjuvant use is an effective strategy to enhance vaccine efficacy. Traditional mineral oil-based adjuvants have been reported with post-immunization reactions. Developing new adjuvant formulations with improved potency and safety will be of great value.

**Results:**

In the study reported herein, a novel oil-in-water (O/W) Emulsion Adjuvant containing Squalane (termed EAS) was developed, characterized and investigated for swine influenza virus immunization. The data show that EAS is a homogeneous nanoemulsion with small particle size (~ 105 nm), low viscosity (2.04 ± 0.24 cP at 20 °C), excellent stability (at least 24 months at 4 °C) and low toxicity. EAS-adjuvanted H3N2 swine influenza vaccine was administrated in mice subcutaneously to assess the adjuvant potency of EAS. The results demonstrated that in mice EAS-adjuvanted vaccine induced significantly higher titers of hemagglutination inhibition (HI) and IgG antibodies than water-in-oil (W/O) vaccines or antigen alone, respectively, at day 42 post vaccination (dpv) (*P* < 0.05). EAS-adjuvanted vaccine elicited significantly stronger IgG1 and IgG2a antibodies and higher concentrations of Th1 (IFN-γ and IL-2) cytokines compared to the W/O vaccine or antigen alone. Mice immunized with EAS-adjuvanted influenza vaccine conferred potent protection after homologous challenge.

**Conclusion:**

The O/W emulsion EAS developed in the present work induced potent humoral and cellular immune responses against inactivated swine influenza virus, conferred effective protection after homologous virus challenge and showed low toxicity in mice, indicating that EAS is as good as the commercial adjuvant MF59. The superiority of EAS to the conventional W/O formulation in adjuvant activity, safety and stability will make it a potential veterinary adjuvant.

## Background

Vaccination is the principal strategy for prevention and control of diseases, and adjuvant use is an effective strategy to enhance vaccine efficacy. Emulsions have a long history as adjuvants for both human and animal vaccines. However, traditional oil-based emulsion adjuvants, such as Freund’s complete or incomplete adjuvant, have been reported with post-immunization reactions. Developing new adjuvant formulations with improved potency and safety will be of great value.

Swine influenza is an acute respiratory disease of pigs mainly caused by the influenza A virus. Although mortality is usually low, swine influenza may result in poor growth, weight loss, immunosuppression of infected pigs and economic loss in the pig industry [1]. Furthermore, it has been hypothesized that pigs can serve as an intermediate host for the adaptation of avian influenza viruses to humans or as mixing vessels for the generation of reassortant viruses [2–4]. Vaccination is the primary strategy for prevention and control of swine influenza in China and other countries. However, previous studies demonstrate that commercial vaccines in a water-in-oil (W/O) formulation is not highly effective in preventing vaccinated pigs from wild-type virus infection [5, 6]. In addition, post-immunization reactions associated with mineral oil-based adjuvants have been reported, such as edema, abscess/granuloma formation or necrosis at injection sites, which render pork unfit for consumption and greatly limits the wide application of these vaccines [7]. Hence, developing new adjuvant formulations with improved potency and safety will be of great value and impact both in the swine industry and for global public health. In this study, swine influenza virus was used as a model antigen to investigate the new adjuvant’s immunogenicity.

The most successful adjuvants of influenza vaccines for human use are squalene-based oil-in-water emulsions, such as MF59 (Novartis) and ASO3 (GlaxoSmithKline). A number of clinical trials indicate that squalene-in-water emulsions outperform aluminum salts or W/O emulsions at increasing vaccine immunogenicity and affording cross-reactivity without causing unacceptable adverse reactions [8–11]. Squalene or squalane (hydrogenated form of squalene) has been used as an alternative to mineral oil for its tolerance and investigated extensively in vaccine adjuvant applications. In the current study, a new squalane-in-water (O/W) emulsion, termed EAS, was developed for veterinary vaccines. We describe the main physicochemical characteristics, stability and tolerability of the new O/W emulsion adjuvant. Then, vaccine supplemented with EAS was used to immunize mice subcutaneously to assess its capacity to induce a specific immune response. Our results show that EAS-adjuvanted H3N2 swine influenza vaccine can induce significantly higher antibody titers than W/O vaccines in mice, and elicit a mixed Th1/Th2 response. Compared to the conventional W/O formulation adjuvant, EAS was shown to be a better candidate for swine influenza immunization due to its superiority in adjuvant efficacy, stability and safety.

## Results

### Characterization of the O/W emulsion EAS

**Fig. 1:**
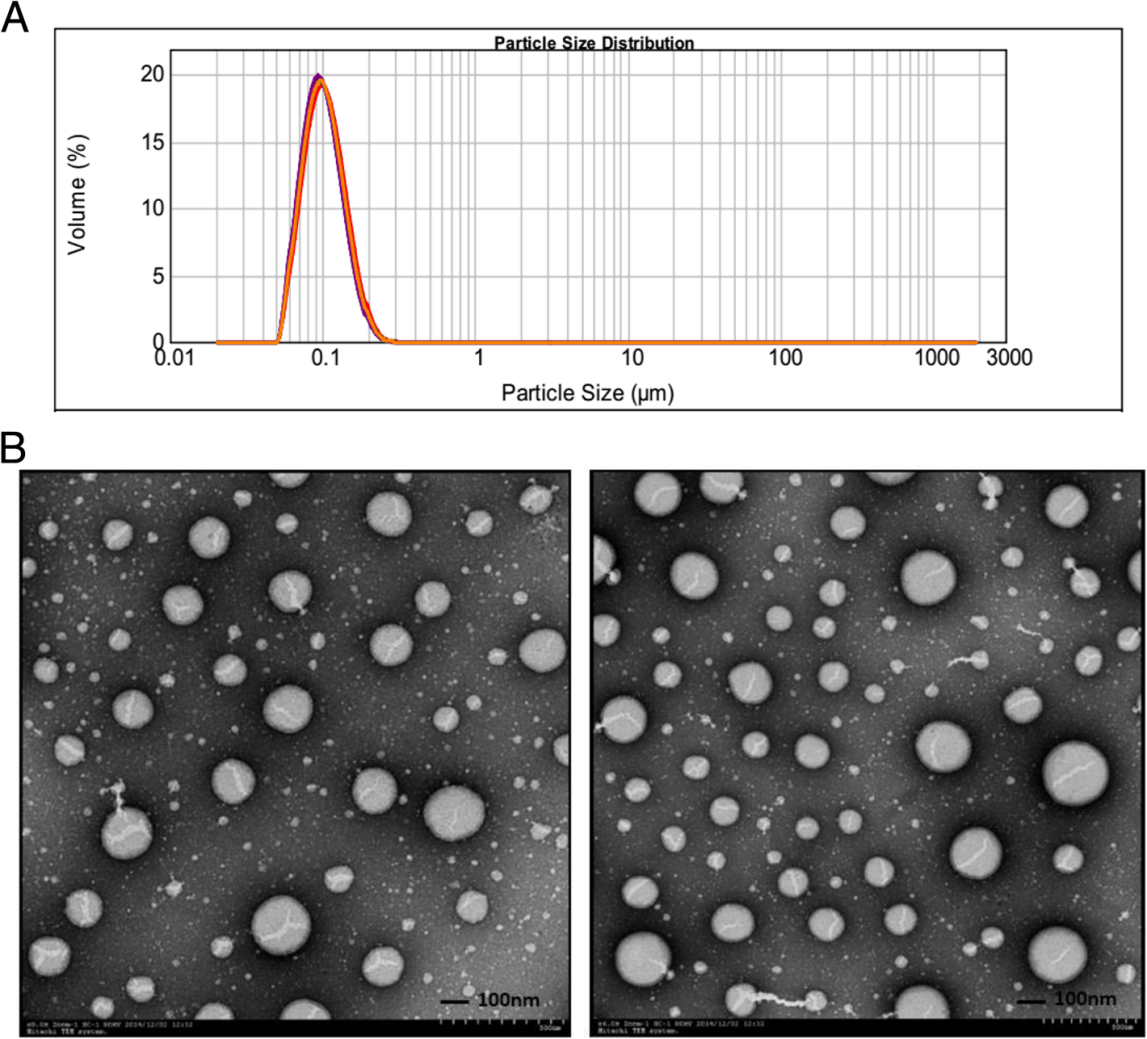
The particle size distribution and TEM images of EAS. **a**: Nine total measurements were made on 3 separate samples from the same batch of EAS as measured by dynamic light scattering. **b**: Cryo-transmission electron microscopy of two samples of EAS. Bar represents 100 nm

A novel emulsion formulation containing squalane, PEG-PPG-PEG, soy lecithin and carbopol was prepared. Particle size, polydispersity index, zeta potential, viscosity, conductivity and pH value of the developed emulsion EAS were measured (Table 1). The mean particle size of the EAS emulsion was ~ 105 nm, with a low polydispersity index (Table 1 and Fig. 1a). TEM analysis was performed to investigate the morphology of emulsion droplets (Fig. 1b). Images from the O/W emulsion showed a characteristic spherical shape, and the diameter of emulsion droplets was in agreement with DLS measurements as described above. The zeta potential value of these emulsions was about − 38 mV, higher than the common slightly negative charge usually reported for other O/W emulsions [12]. EAS had a viscosity of 2.04 ± 0.24 cP at 20 °C. Conductivity measurements demonstrated that EAS is an oil-in-water system [13].

**Table 1:** Characteristics of the O/W emulsion adjuvant (values are shown as means± SD, *n* = 3)

Visual appearance	Particle size (Dv50; nm)	Polydispersity index (PDI)	Zeta potential (mV)	Viscosity (cP)	Conductivity (mS/cm)	pH
Milky-white liquid	105 ± 2	0.12 ± 0.03	− 38.8 ± 0.2	2.04 ± 0.24	−0.0269 ± 0.0017	6.80 ± 0.02

### Stability of the O/W emulsion EAS

The stability of EAS at different storage temperatures was monitored by visual inspection and particle sizing change. At 4 °C, the emulsion had minimal changes in particle size, zeta potential, pH value and viscosity over 24 months (Table 2). At elevated temperatures (25 or 37 °C) the samples demonstrated stability over 6-month or 3-month durations, respectively. The samples stored at 25 or 37 °C had an increase in particle size after 6 months or 3 months, respectively. Thereafter, visible evidence of coalescence or phase separation of the oil and aqueous phases gradually emerged. Overall, results provided in Table 2 support the long-term stability of EAS at 4 °C.

**Table 2:** Heat Stability Study of EAS at 4 °C, 25 °C and 37 °C (values are shown as means ±SD, *n* = 3)

EAS		Time (months)							
		T0	1	3	6	9	12	18	24
4 °C	Particle size	104 ± 2	104 ± 2	101 ± 3	105 ± 1	104 ± 2	105 ± 2	107 ± 2	106 ± 2
	Zeta potential	−38.8 ± 0.1	−38.3 ± 0.4	− 38.5 ± 0.2	− 38.8 ± 0.2	− 38.7 ± 0.2	−38.5 ± 0.2	− 38.7 ± 0.02	−38.3 ± 0.3
	pH	6.8 ± 0.1	6.7 ± 0.2	6.8 ± 0.1	6.6 ± 0.3	6.8 ± 0.1	6.8 ± 0.1	6.7 ± 0.3	6.7 ± 0.2
	Viscosity	2.01 ± 0.14	2.01 ± 0.21	1.90 ± 0.17	1.94 ± 0.17	2.04 ± 0.15	2.11 ± 0.01	1.99 ± 0.04	2.03 ± 0.12
25 °C	Particle size	104 ± 2	103 ± 3	109 ± 1	114 ± 2	141 ± 9	ND	ND	ND
	Zeta potential	−38.3 ± 0.1	−38.4 ± 0.5	−38.7 ± 0.3	−38.5 ± 0.5	−35.4 ± 0.4	ND	ND	ND
	pH	6.8 ± 0.2	6.5 ± 0.3	6.8 ± 0.1	6.5 ± 0.3	6.6 ± 0.2	ND	ND	ND
	Viscosity	2.01 ± 0.05	2.04 ± 0.01	2.24 ± 0.23	2.17 ± 0.05	2.43 ± 0.22	ND	ND	ND
37 °C	Particle size	105 ± 2	107 ± 2	115 ± 2	157 ± 12	ND	ND	ND	ND
	Zeta potential	−38.6 ± 0.3	−38.4 ± 0.2	−36.9 ± 0.3	−35.8 ± 0.4	ND	ND	ND	ND
	pH	6.8 ± 0.2	6.5 ± 0.3	6.6 ± 0.2	6.7 ± 0.2	ND	ND	ND	ND
	Viscosity	2.03 ± 0.11	2.11 ± 0.18	2.34 ± 0.19	2.55 ± 0.38	ND	ND	ND	ND

### Toxicity evaluation of EAS

**Fig. 2:**
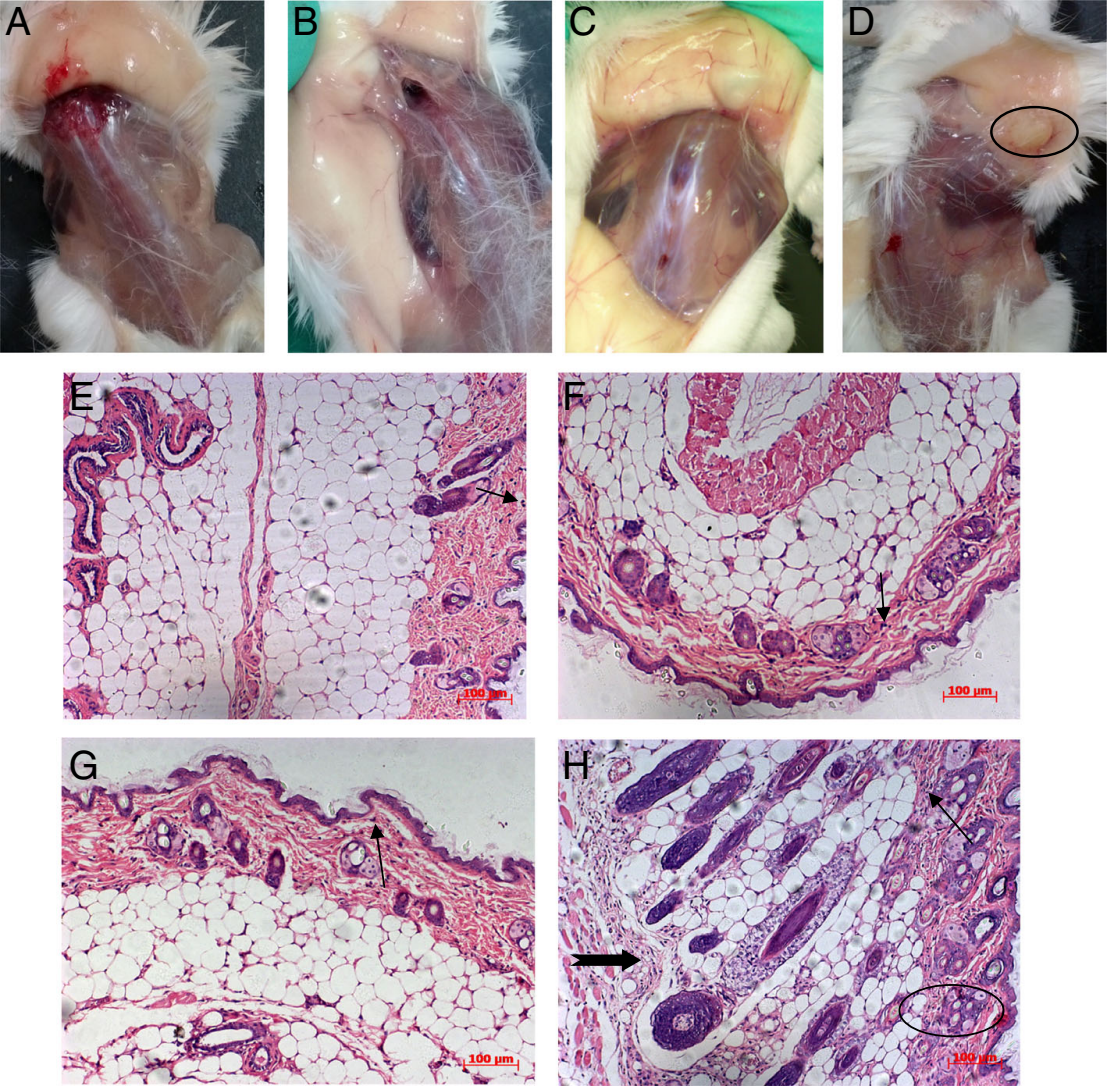
Toxicity analysis of EAS. **a** to **d**: the local reactogenicity of different formulations at the sites of injection. The subcutaneous injection sites of each formulation in female BALB/c mice were observed 72 h after administration. **a**: PBS control; **b**: EAS; **c**: MF59; **d**: W/O formulation. **e** to **h**: the histology of the subcutaneous injection sites of mice (H.E. × 100).The tissues excised from the sites of administration 72 h after the injection of each sample were fixed in 10% formalin and embedded in paraffin for histologic examination. Hematoxylin and eosin stained slides were prepared using standard methods. **e**: PBS control; **f**: EAS; **g**: MF59; **h**: W/O formulation

To assess EAS tolerability in animals, the local reactogenicity of different formulations was monitored at injection sites for edema or erythema and animals were observed for health problems following injection. There were not any changes at the sites of injection in the PBS group (Fig. 2a). No systemic effects or gross lesions were noted post-administration of the EAS and MF59 formulations (Fig. 2 b and c). When administered with the W/O formulation, mild to serious reactions, such as edema, abscesses, granulomas and nodules were present at injection sites (Fig. 2d).

Histological analysis was used to evaluate the toxicity of the different formulations. No pathologic changes were observed in control group (Fig. 2e), whereas inflammation, as evidenced by hyperplasia of connective tissue (thick arrow), folliculus pili and hair papilla (ellipse); by infiltrating PMNs (thin arrow) were present in tissue after injection with W/O emulsion (Fig. 2h). The pathologic changes in the EAS and MF59 emulsion groups were mild and there were no significant differences between these two groups (Fig. 2 f and g).

### EAS-adjuvanted vaccine induces a high serum HI antibody response

**Fig. 3:**
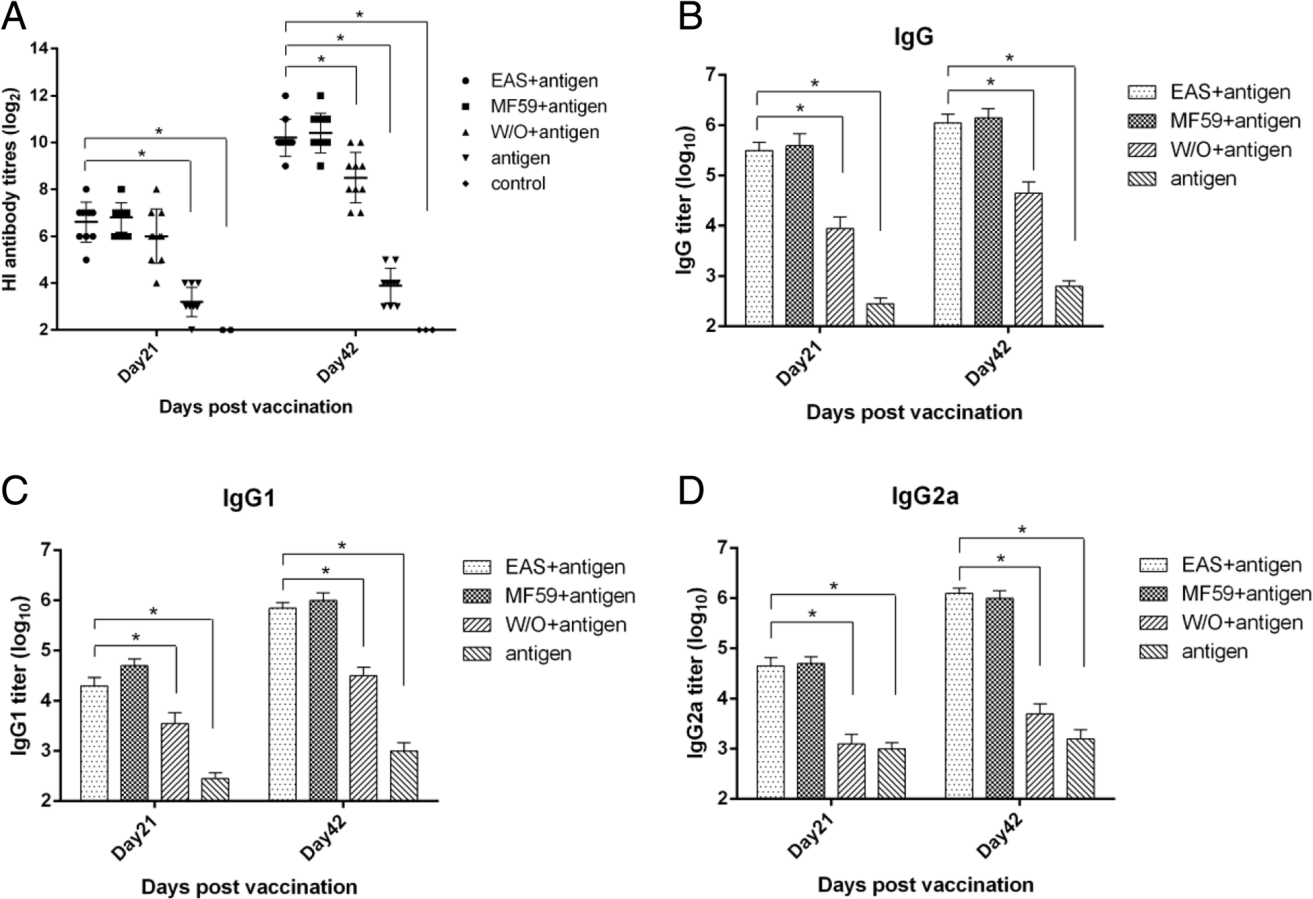
HI antibody titers and antigen (A/swine/Shandong/3/2005)-specific antibodies (IgG, IgG1 and IgG2a) in BALB/c mice after immunization with various formulations. Mice received two immunizations at 3 weeks intervals and sera were collected on days 21 and 42 after primary immunization. Data are shown as individual data points or means ±SD. Asterisks indicate statistically significant differences (*P* < 0.05) between groups

Mice were immunized with swine influenza antigen incorporated into different adjuvants. Sera samples were collected 3 weeks after the first and second immunization. The haemagglutination inhibition (HI) assay was used to evaluate the anti-HA antibody response. As shown in Fig. 3a, all adjuvant formulations induced significantly higher HI titers than the non-adjuvanted vaccine group at days 21 and 42 post-vaccination (dpv) (*P* < 0.05)). EAS induced significantly higher HI titers than the W/O formulation at 42dpv (*P* < 0.05)), although there were no statistically significant difference between them at 21dpv (*P* > 0.05). Minimal differences in antibody responses were observed between animals immunized with EAS and MF59 adjuvant at 21 and 42dpv (*P* > 0.05).

### EAS-adjuvanted vaccine induces high titers of humoral immune reactive antibody

Humoral immune responses induced by different formulations were detected by measuring H3N2 specific IgG antibodies of vaccinated mice using ELISA. All adjuvant formulations elicited significantly higher total IgG titers compared to antigen alone at both 21 and 42dpv (*P* < 0.05, Fig. 3b). EAS and MF59 vaccinated groups had significantly higher total IgG titers than the W/O formulation at 3 weeks after the first and second immunizations (*P* < 0.05). But there were no statistical differences between EAS and MF59 vaccinated groups (*P* > 0.05). Anti-flu IgG1 and IgG2a serum levels were determined to indicate the different qualitative response types induced by various formulations. As shown in Fig. 3c, all adjuvant formulations significantly increased antigen-specific IgG1 antibodies compared to antigen alone both at 21 and 42dpv (*P* < 0.05). However, only EAS- or MF59-adjuvanted antigen significantly amplified IgG2a responses compared to the W/O formulation or antigen alone after primary or booster immunization (*P* < 0.05, Fig. 3d).

### EAS-adjuvanted vaccine elicits high concentrations of cytokines and a mixed Th1/Th2 profile

**Fig. 4:**
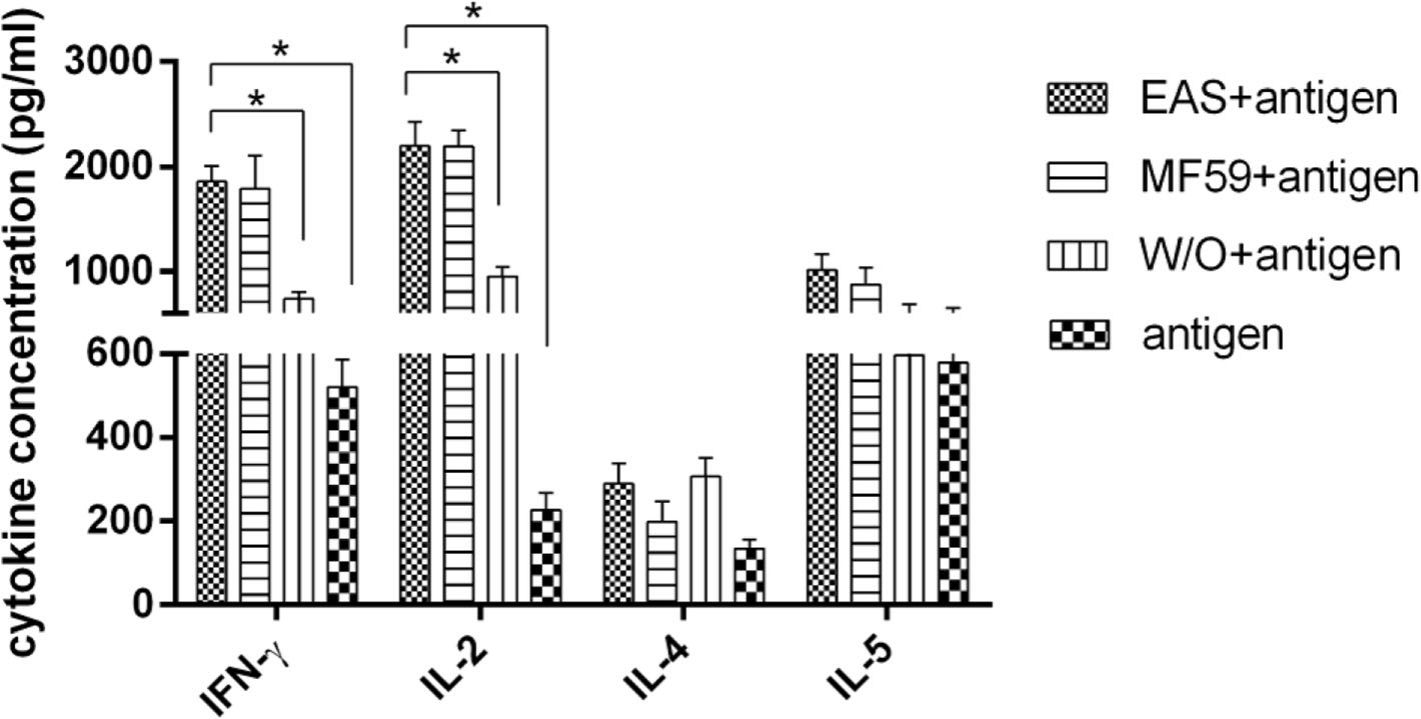
The cytokine response induced in the spleen of BALB/c mice after immunization with various formulations. Mice (*n* = 3) received 2 immunizations at 3 weeks intervals and splenocytes were collected at day 21 after booster immunization. The cell supernatant was analyzed for influenza-induced IFN-γ, IL-2, IL-4 and IL-5 by radioimmunoassay after 48 h of stimulation. Data are shown as means ± SD. Asterisks indicate statistically significant differences (*P* < 0.05) between groups

The concentration of cytokines in supernatants of ex vivo stimulated splenocytes was measured by radioimmunoassay. No or very low levels of cytokines were detected in the supernatant of splenocytes from control mice. Splenocytes from mice immunized with EAS-adjuvanted vaccine secreted significantly higher levels of Th1 cytokines (IFN-γ and IL-2) compared to the W/O formulation or antigen alone group 48 h after stimulation (*P* < 0.05, Fig. 4). Additionally, higher levels of Th2 cytokines (IL-4 and IL-5) were detected in the EAS-adjuvanted vaccine group compared to the other vaccine groups, although there was no significant difference between them (*P* > 0.05). Cytokine profiles after 48 h of stimulation were similar between EAS and MF59 vaccine groups.

### EAS-adjuvanted vaccine confers effective protection against homologous virus challenge

**Fig. 5:**
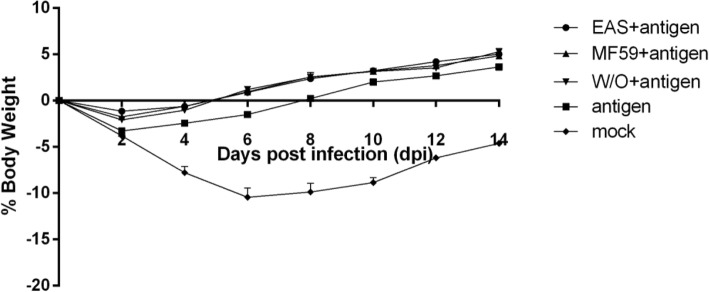
Weight changes of mice after homologous virus challenge. Mice were challenged intranasally with 0.2 × 10^5^ EID50 of the H3N2 subtype virus strain A/swine/Shandong/3/2005 two weeks after the second immunization. The survival rate, clinical signs and bodyweight of the challenged mice were monitored for 14 days after the challenge. Data are shown as means ± SD. Asterisks indicate statistically significant differences (*P* < 0.05) between groups

All mice survived and no obvious clinical signs were observed after challenge. Virus was not detected in the lungs of immunized mice at 5 dpi except the antigen alone vaccinated group or mock vaccinated group (Table 3). The immunized mice in EAS, MF59 and W/O group showed slight and transient body weight loss (1%~ 2%) on 2 dpi, and then recovered quickly from 4 dpi. In antigen alone immunized group, mice showed mild weight loss (less than 5%), then they recovered gradually and regained their body weight on 8 dpi. In contrast, mock vaccinated mice displayed significant and progressive weight loss, which lasted 2 weeks, and the worst weight loss with 10.5% decline occurred on 6 dpi (Fig. 5).

**Table 3:** Lung virus isolation from mice of different groups challenge with the homologous virus

Group	Number of mice carrying H3N2 virus in lung post challenge/total number (Positive rate)
EAS + antigen	0/5
MF59 + antigen	0/5
W/O + antigen	0/5
antigen	1/5
mock	4/5

Mice were challenged intranasally with 0.2 × 10^5^ EID50 of the H3N2 subtype virus strain A/swine/Shandong/3/2005 two weeks after the second immunization. Five mice of each group were euthanized at 5 days post infection (dpi), and whole lungs were collected and homogenized in PBS. The homogenates were centrifuged and the supernatant from each sample was collected for passage in 10-day-old specific pathogen free (SPF) embryonated chicken eggs. The allantoic fluid was harvested and tested for hemagglutinin activity

## Discussion

For farm and laboratory animals, the most commonly used emulsion adjuvants are mineral (paraffin) oil-based Freund’s complete or incomplete adjuvant. Although these W/O emulsions have adjuvant effects in veterinary vaccines, they are too reactogenic for routine use [14]. O/W emulsion, owing to the generally lower concentration of oils and better safety and tolerability profiles, will likely be preferred for the next generation of adjuvants. The objective of this study was to develop a novel O/W emulsion and investigate its adjuvant potential in inactivated swine influenza vaccine. DLS and TEM analyses document that EAS is a homogeneous nanoemulsion with small particle size (~ 105 nm) and a low polydispersity index (0.159 ± 0.036). The low viscosity (2.04 ± 0.24 cP at 20 °C) was expected because of the small average diameter of the droplets. The small average diameter and low viscosity of EAS will be of benefit following sterile filtration and parenteral administration.

The adjuvant potential of EAS was investigated and compared with the W/O formulation and MF59 adjuvant in mice immunized with inactivated swine influenza virus. The results showed that mice immunized subcutaneously twice with EAS-adjuvanted vaccine had significantly higher titers of HI and IgG antibodies than the W/O formulation or antigen alone (*P* < 0.05), but there was no difference between the EAS-adjuvanted group and MF59-adjuvanted group (*P* > 0.05). Remarkably, data from many previous studies indicates that the size of adjuvant particles has an important effect on adjuvant activity and the type of evolving immune responses. For example, Li et al. showed that smaller OVA-conjugated nanoparticles (~ 230 nm) induced stronger OVA-specific antibody and cellular immune responses than the larger OVA-nanoparticles (~ 780 nm) when subcutaneously injected into mice [15]. Foged et al. [16] and Kanchan et al. [17] also showed that smaller particles (< 500 nm) were more efficiently internalized by DCs and macrophages than larger particles (> 1 μm). Moreover, the particle size of MF59 (~ 160 nm) was optimized in the preparation process by microfluidizer and was crucial to its maximal adjuvant effect for a wide range of antigens in many clinical and pre-clinical studies [18]. Since the mean particle size of EAS was less than 200 nm, we presume that it may have similar effect on its adjuvant efficacy. The correlation between the particle size of EAS and its adjuvant activity will be defined in further studies.

Immunity to influenza is dependent on both antibody and cell mediated immune responses, and both are critical to inducing protective immunity [19–22]. Oil in water adjuvants including MF59 and AS03 were shown to significantly improve humoral and T cell responses against human or avian influenza [8–11]. In the current study, although all vaccinated mice induced amplified H3N2 specific ELISA antibodies, only the EAS and MF59 vaccinated groups induced both strong IgG1 and IgG2a antibodies, while the W/O formulation or antigen alone induced predominantly IgG1 antibodies. The predominance of IgG2a isotypes indicates skewing towards Th1 responses, which promotes efficient generation of cytotoxic T cells resulting in faster virus clearance and recovery after viral infection [23–25]. These results demonstrate that EAS induces a balanced IgG1/IgG2a response and a mixed Th1/Th2 response, which is important for protective immunity against influenza. Furthermore, the cytokine profile from the supernatants of ex vivo stimulated cells showed that the EAS-adjuvanted vaccine induced significantly higher levels of Th1 cytokines compared to the W/O formulation or antigen alone, implying an effective stimulation of the innate immune system and consequent facilitation of enhanced immunity to influenza, as did MF59. Moreover, no virus was detected in the lungs of immunized mice at day 5 post infection except the antigen alone vaccinated group or mock vaccinated group, showing that EAS-adjuvanted vaccine, as well as MF59-adjuvanted vaccine and the conventional W/O vaccine could provide effective protection against homologous challenge. Taken together, EAS-adjuvanted vaccine enhanced HI titers, induced virus-specific cell-mediated immune response and provided potent protection against virus challenge. EAS could be a promising candidate adjuvant for veterinary vaccine. Remarkably, although the adjuvant effect of MF59 was due to the fully formulated emulsion [18, 26, 27], we presume that some component of EAS might induce a comparable adjuvant effect. Specifically, carbopol-based adjuvant suspensions have been evaluated in veterinary vaccines against several pathogens [28, 29], and the non-ionic block polymer pluronic® L121 (similar to pluronic® L31 in chemical structure) has been reported to contribute to the potent adjuvant efficacy of the Syntex adjuvant formulation (SAF) [30, 31]. We presume that these ingredients may account for the enhanced immune response of the EAS formulation. Research on the mechanism of action of EAS is ongoing.

For an emulsion to be used for parenteral administration, stability is one of the most important parameters. Many factors are associated with the stability of emulsions, such as initial particle size, uniformity, surface tension and interface charge [32–34]. Emulsions with smaller initial particle size are more stable than those with larger particles [35]. For instance, the small droplet size of MF59 emulsion enhances emulsion stability for at least 2 years at 2–8 °C [26, 36]. Moreover, higher zeta potential is closely related to a higher surface charge of colloidal particles and thus has more charge repulsion, which is another indication of the stability of colloidal systems [35, 37]. The zeta potential of EAS was about − 38 mV, significantly higher than the common value of − 20 to − 30 mV reported for other O/W emulsions [37]. This might be due to the use of soy lecithin and carbopol 971P, which contains various anionic fractions. These negatively charged components usually account for a major proportion of surface charge and hence are of great importance to the stability of emulsions. In the current study, EAS was stable for 24 months and did not present any significant physical alteration or phrase separation over the storage period at 4 °C. We presume that the small particle size, low polydispersity index and high zeta potential may contribute to its excellent stability.

Beside the potent adjuvant efficacy and superior stability of EAS, safety is another major concern when considering new adjuvant formulations for vaccine applications. The local reactogenicity and tolerability of EAS was assessed in mice by visual inspection and histological analysis. Although mild inflammatory reactions were present in mice injected with EAS, there were no obvious health problems or gross lesions. However, mice injected with the W/O formulation, had adverse reactions at injection sites. The safety profile of EAS should be further tested in pigs or other farm animals in the near future. Moreover, during pandemic, it is very often that antigen is in short and it is important to have an adjuvant to save lots of antigens. Thus, dose-dependent tests are needed. And, it will be also nice to examine whether this adjuvant enhances long-term protection, cross-protection of vaccine and whether the adjuvant will enhance the immunity itself, and so on. These will be investigated in the further study.

## Conclusion

Developing new adjuvant formulations with improved potency, enhanced safety and low viscosity will be of great value for swine industry. The O/W emulsion EAS developed in the present work induced potent humoral and cellular immune responses against inactivated swine influenza virus, conferred effective protection after homologous virus challenge and showed low toxicity in mice, indicating that EAS is as good as the commercial adjuvant MF59. The superiority of EAS to the conventional W/O formulation in adjuvant activity, safety and stability will make it a potential veterinary adjuvant.

## Methods

### Adjuvant components and virus

Squalane (vegetable-based) was purchased from Croda Ltd., Shanghai, China. Polyoxyethylene 20 sorbitan monooleate (polysorbate 80) and Poloxamer 407 were purchased from Sigma-Aldrich (St. Louis, MO, USA). Poly (ethylene glycol) -block-poly (propylene glycol) -block-poly (ethylene glycol) (PEG-PPG-PEG Pluronic® L-31) was obtained from BASF Corporation, USA. Soy lecithin (PC~ 70%) was purchased from Shanghai Tywei Pharmaceutical Co., Ltd. (Shanghai, China). Polyethylene glycol 400 monooleate (PEG400MO) was purchased from Xinchang pharmaceutical Co., Ltd. (Zhejiang, China). Carbomer 971P was obtained from Lubrizol (Wickliffee, OH, USA). A/swine/Shandong/3/2005 (H3N2 subtype) was isolated from pigs with respiratory disease on farms in eastern China [38]. Viruses were propagated in the allantoic cavities of 10-day-old embryonated specific-pathogen-free (SPF) chicken eggs or in Madin-Darby canine kidney cells (MDCK). The infective allantoic fluid was aliquoted and stored at − 80 °C.

### Preparation of emulsion

The recipe of the emulsion adjuvant was as described in the Chinese patent license with registration number 201310021011.3. Briefly, the lipophilic emulsifiers, PEG-PPG-PEG Pluronic® L-31 and soy lecithin were fully dissolved in the oil phase (squalane) and the hydrophilic emulsifiers, polysorbate 80, Poloxamer 407, and PEG400MO were fully dissolved in the water phase (PBS, pH 7.4) containing carbopol 971P. The oil phase was added to the aqueous phase with a high-shear mixer (MICCRA D-9, Nordrhein-Westfalen, Germany) at 11,000 rpm for 15 min to yield a coarse emulsion. The coarse emulsion was immediately processed through a High Pressure Homogenizer (ATS engineering, Shanghai, China) for six cycles at 1000 bar. Finally, the emulsion was filter-sterilized by 0.22 μm filtration (Millipore AS, Oslo, Norway) and adjusted to pH 6.8. Emulsion adjuvant containing squalane was named EAS.

### Characterization of emulsion

Emulsion particle sizing was determined via dynamic light scattering (DLS) with a Mastersizer 2000 (Malvern Instruments, Worcestershire, UK). The size and homogeneity of oil droplets in EAS were observed by transmission electron microscopy (TEM) with a JEM-1230 (JEOL Ltd., Tokyo, Japan), as described previously [39]. Zeta potential was determined on a Zetasizer Nano-ZS 90 (Malvern Instruments Corp., UK). The dynamic viscosity of EAS was measured at 25 °C using a digital viscometer (DV-II + Pro, Brookfield Engineering Laboratory, Middleboro, MA, USA).

For stability analysis, filter sterilized EAS samples were stored at 4, 25 and 37 °C over 24 months. Colloidal stability of emulsion was assessed by visual inspection and particle sizing change. Laser light diffraction techniques were used for size measurements because of their wide range of particle sizing, enabling the detection of larger droplets that may appear as a result of emulsion destabilization.

### Preparation of vaccine

The H3N2 subtype swine influenza vaccine was prepared as an O/W form. In brief, the H3N2 subtype virus strain A/swine/Shandong/3/2005 was propagated in allantoic cavities of 10-day-old specific pathogen free (SPF) embryonated chicken eggs. The viral-laden allantoic fluids (1024 HA units per 0.1 ml) were purified by centrifugation (28,000×g, 30 min, 4 °C) and inactivated with 0.5% (*v*/v) β-propiolactone at 37 °C for 24 h. Equal volumes of purified virus and EAS were thoroughly mixed on a shaker to manufacture an O/W emulsion vaccine. MF59 emulsion adjuvant and MF59-adjuvanted vaccine was also prepared according to the protocol published for MF59 [26]. Briefly, MF59, consisting of 5% squalene (v/v), 0.5% Tween80 (v/v), 0.5% Span 85 (v/v) (Sigma, St. Louis, MO, USA) in 20 mM citrate buffer, was prepared by homogenization at 12,000 psi with a Microfluidizer (model 110Y; Microfluidics, Newton, MA). The emulsion was sterilized by 0.22 μm filtration. MF59-adjuvanted vaccine was prepared by thoroughly mixing of purified virus and MF59 at a ratio of 1:1 (v/v). Conventional vaccine in a water-in-oil form (W/O vaccine) was prepared as previously described and used as a control [40]. For W/O emulsion vaccine, the purified virus was emulsified in Marcol 52 mineral oil (ESSO, Paris, France) at a ratio of 1:3 (v/v, virus to adjuvant).

### Toxicity analysis

Female BALB/c mice (18-22 g) purchased from Beijing Merial Vital Laboratory Animal Technology Co., Ltd. (Beijing, China) were housed under specific pathogen-free conditions. Mice from each group (*n* = 3) were inoculated subcutaneously with 200 μl of EAS, MF59 or W/O emulsion. Mice in the control group received the same amount of PBS. The tissues excised from the sites of administration 72 h after injection were fixed in 10% formalin and embedded in paraffin for histologic examination. Hematoxylin and eosin stained slides were prepared using standard methods [41].

### Immunization of mice

Female BALB/c mice (18-22 g) purchased from Beijing Merial Vital Laboratory Animal Technology Co., Ltd. (Beijing, China) were housed under specific pathogen-free conditions. Mice from each group (*n* = 20) were injected subcutaneously twice with 200 μl of O/W emulsion vaccines (containing 1024 HA units) or 400 μl of W/O vaccine (containing 1024 HA units) at 3 weeks intervals. Mice in the control group received immunizations with 200 μl of PBS. Sera were collected 3 weeks after the first and second immunization.

### Hemagglutination inhibition titer (HI)

To determine hemagglutination-inhibition (HI) titers, sera were first treated with receptor-destroying enzyme (RDE) (DenkaSeiken, Tokyo, Japan) by incubation overnight at 37 °C to eliminate hemagglutination non-specific inhibitors in serum samples. Then, sera were heated at 56 °C for 30 min to inactivate the receptor-destroying enzyme. Sera were serially (1:2) diluted, mixed with 4HA units of influenza virus, and incubated for 30 min at room temperature prior to adding 1% chicken red blood cells. HI titers are defined as the highest serum dilution completely inhibiting hemagglutination.

### Elisa

Influenza virus-specific antibodies (IgG, IgG1 and IgG2a) were quantified using an ELISA assay, as previously described [42] except that plates were coated with inactivated whole H3N2 influenza virus antigen (2 μg/ml). The titers of IgG, IgG1 and IgG2a were calculated using a four-parameter logistic equation (Softmax software, Molecular Devices). The inflection point of the titration curve was taken as titer value.

### Ex vivo re-stimulation of splenocytes

Antigen-specific T cell responses were measured following re-stimulation of splenocytes with whole β-propiolactone-inactivated H3N2 influenza virus. Briefly, splenocytes were isolated from immunized mice and cultured in RPMI-1640 supplemented with 10% fetal bovine serum, 100 U/ml penicillin, and 0.1 mg/ml streptomycin. Splenocytes (2 × 10^6^cells/well) were then stimulated with inactivated H3N2 influenza virus (2 μg/ml). Forty-eight hours later, cytokines (IL-2, IL-4, IL-5, IFN-γ) were measured from supernatants by radioimmunoassay. Commercial kits were purchased from the Institute of Radiation of Science and Technology Development Center of the General Hospital of People’s Liberation Army (Beijing, China). The assay was conducted according to the manufacturer’s protocol. All samples were assayed in triplicate.

### Virus challenge of immunized mice

Mice were challenged intranasally with 0.2 × 10^5^ EID50 of the H3N2 subtype virus strain A/swine/Shandong/3/2005 two weeks after the second immunization. Five mice of each group were euthanized at day 5 post infection (dpi), and whole lungs were collected and homogenized in PBS. The homogenates were centrifuged and the supernatant from each sample was collected for passage in 10-day-old specific pathogen free (SPF) embryonated chicken eggs. The allantoic fluid was harvested and tested for hemagglutinin activity. The survival rate, clinical signs and bodyweight of the remaining mice were monitored for 14 days after the challenge.

### Statistical analyses

Data were expressed as individual data points or means ± standard deviation (SD). Differences were evaluated by one-way analysis of variance (ANOVA) followed by post-hoc tests. Differences were considered significant at a *P*-value < 0.05.

### Ethics statement

Protocols involving mice were approved by the Institutional Animal Care and Use Committee and were conducted following the guidelines of the Institutional Biosafety Committee at the Jiangsu Academy of Agriculture Sciences. Efforts were made to minimize animal suffering. All the animals were euthanized after the study in a chamber with carbon dioxide (BIOSCAPE, CO_2_ – box model L). The animals were placed into CO_2_ – box and 100% carbon dioxide was imported. The following ratios of CO_2_/O_2_% vol/min were applied for induction 5/95% vol/min and for euthanasia 100/0% vol/min. The secondary physical method of euthanasia was performed by decapitation. In the progress, the animals were unconscious.

## Abbreviations

DLS: Dynamic light scattering
dpv: Days post-vaccination
EAS: Emulsion Adjuvant containing Squalane
HI: Hemagglutination inhibition titer
MDCK: Madin-Darby canine kidney
O/W: Oil in water
PEG400MO: Polyethylene glycol 400 monooleate
PEG-PPG-PEG: Poly (ethylene glycol) -block-poly (propylene glycol) -block-poly (ethylene glycol)
polysorbate 80: Polyoxyethylene 20 sorbitan monooleate
RDE: Receptor-destroying enzyme
SD: Standard deviation
SPF: specific-pathogen-free
TEM: Transmission electron microscopy
W/O: Water in oil
